# Supplementary material for: Functionalization with Polyphenols of a Nano-Textured Ti Surface through a High–Amino Acid Medium: A Chemical–Physical and Biological Characterization
Source: Nanomaterials (Basel). 2022 Aug 24;12(17):2916. doi: 10.3390/nano12172916 (PMC9458157; doi:10.3390/nano12172916)
Supplement: Supplementary file 1 [file nanomaterials-12-02916-s001.zip › nanomaterials-1837591-supplementary.pdf]

Fourier-transform infrared spectroscopy (FTIR-ATR)

FTIR-ATR spectra of the samples before and after functionalization are reported in Figure S1. The spectrum of Ti-MP is characterized by a broad band around 823 cm^{-1}, which is attributed to Ti–O bonds [1,2]. Bands at about 1097 cm^{-1} and 1660 cm^{-1} are assigned to carbon-related contaminants (C–OH bond) and adsorbed water, respectively [3,4].

The FTIR-ATR spectrum of Ti-Nano is reported in the same Figure. Some changes in the peaks occur due to the formation of a thin oxide layer on the surface. Differently from other chemical surface treatments reported in the literature on Ti and Ti alloys [5], in this case the broad peak in the region around 3500–300 cm^{-1} related to surface OH groups is absent.

The control surface soaked in the medium without polyphenols (Ti-Nano/HP) and the one functionalized with polyphenols in a high-amino acid medium give very similar spectra and it can be concluded that this technique is not suitable for evidencing the presence of polyphenols on the surfaces, if other similar organic compounds are also adsorbed, such as amino acids. This is expected considering that the functional groups of amino acids and polyphenols are very similar: peaks at 1633 cm^{-1} can be referred to OH bending, at 1620 cm^{-1} (C=O stretching), 1531 cm^{-1} (assigned to C–C aromatic rings), 1599 cm^{-1} (C=C), 1514 cm^{-1} (B-ring) and 1263 cm^{-1} (C–OH phenolic oxydril group) [6,7]. Besides these bands, it is possible to identify a broad band in the area around 3300 cm^{-1}, assigned to O–H stretching vibrations [8,9] and another band in the area around 1300 cm^{-1}, which corresponds to a combination of O–H deformation vibrations and C–O stretching vibrations [10].

In conclusion, FTIR spectra do not give important information on the samples functionalized in the high-amino acid medium because of the large variety of similar compounds adsorbed on the surface.

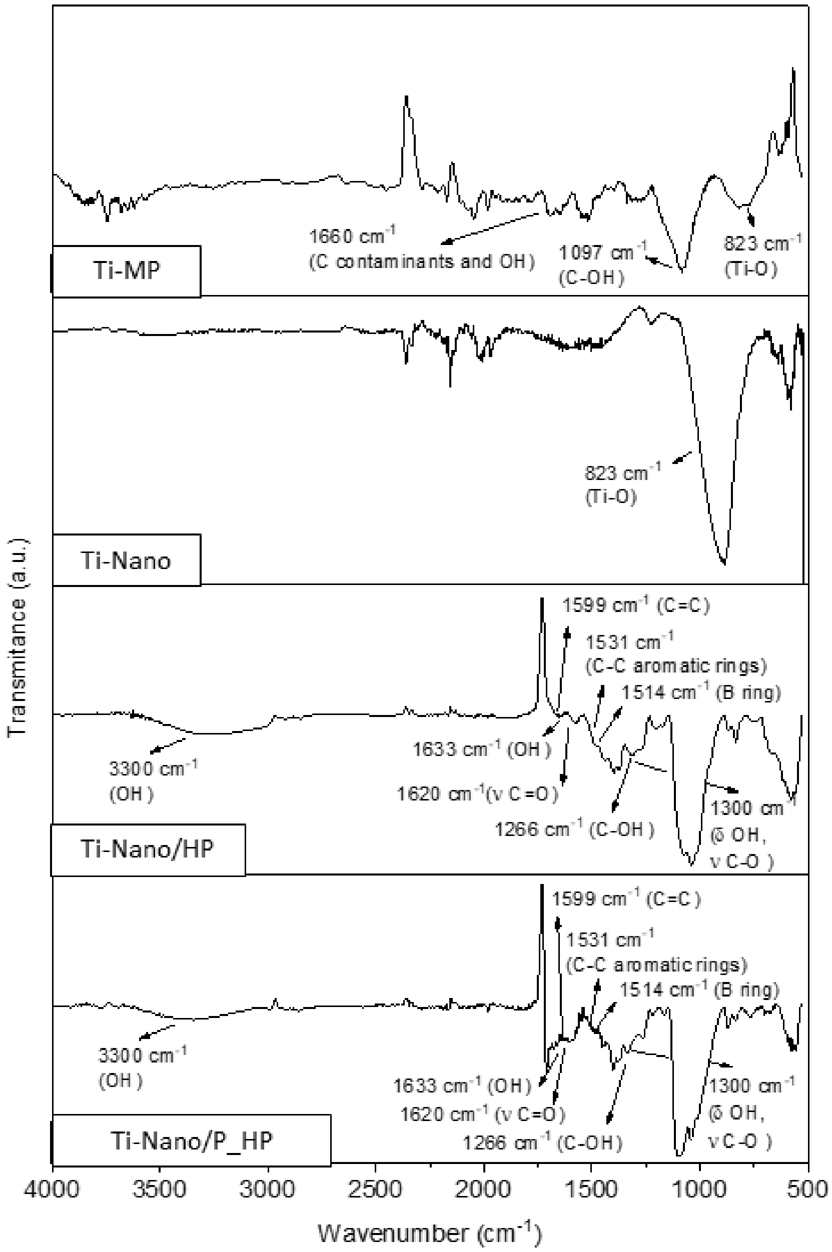

Figure S1. FTIR spectra of Ti-MP, Ti-Nano, Ti-Nano/HP, Ti-Nano/P_HP1.

A high-resolution image of the stained UMR-106 cell cultures is reported in Figure S2. The qualitative results for the Alizarin Red staining were similar to the staining pattern observed for primary osteogenic cells, isolated by sequential trypsin/collagenase digestion of calvarial bone from newborn Wistar rats, and grown on a titanium surface structured at the micron and nanoscale [11]. Even on a transparent, flatter surface (Thermanox coverslips), denser areas of matrix mineralization appear deep dark stained under transmitted light microscopy [12]; on a white material surface (Y-TZP + 64S bioglass), Alizarin Red-stained UMR-106 cultures exhibit a reddish appearance more evidently [13]. No significant differences, in terms of Alizarin Red extraction/calcium content, could be due to the high osteogenic potential of the specific cell culture here used. In this context, the molecular effects observed on differentiated osteoblasts could likely result in a higher impact on bone matrix formation in a cell culture model with lower osteogenic potential, which might therefore be a subject for further investigation.

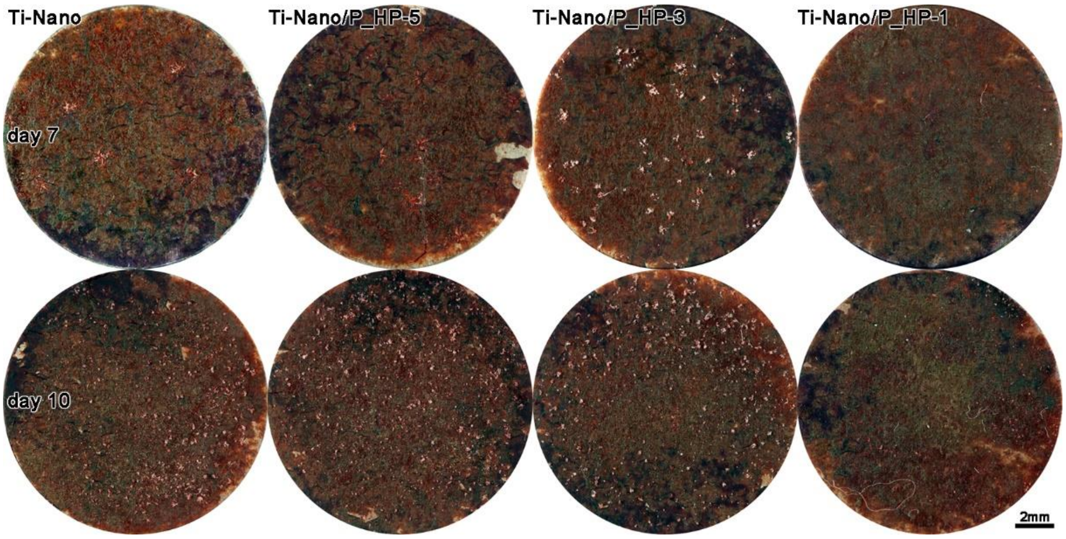

Figure S2. High-resolution imaging of the Alizarin Red staining of UMR-106 cell cultures grown on Ti-Nano functionalized with 1·10−5, 1·10−3, 1 mg/mL grape pomace extract and the control surface (Ti-Nano, Ti-Nano/P_HP-5, Ti-Nano/P_HP-3, Ti-Nano/P_HP1) on days 7 and 10 of culture. Cultures were stained with Alizarin Red for the detection of calcium deposits in areas of mineralized matrix formation, which were randomly distributed throughout the entire disk surface (scale bar = 2 mm). The 1 mg/mL group showed a tendency toward greater calcium content on day 7 of culture.

References

1. Watson, S.; Beydoun, D.; Scott, J.; Amal, R. Preparation of nanosized crystalline TiO_2 particles at low temperature for photocatalysis. *J. Nanoparticle Res*. **2004**, 6, 193–207. https://doi.org/10.1023/B:NANO.0000034623.33083.71.
2. Nakamura, M.; Aoki, T.; Hatanaka, Y.; Korzec, D.; Engemann, J. Comparison of hydrophilic properties of amorphous TiO_x films obtained by radio frequency sputtering and plasma-enhanced chemical vapor deposition. *J. Mater. Res*. **2001**, *16*, 621–626. https://doi.org/10.1557/JMR.2001.0089.
3. Davis, K. M.; Tomozawa, M. An infrared spectroscopic study of water-related species in silica glasses. *J. Non. Cryst. Solids* **1996**, *201*, 177–198. https://doi.org/10.1016/0022-3093(95)00631-1.
4. Urlaub, R.; Posset, U.; Thull, R. FT-IR spectroscopic investigations on sol–gel-derived coatings from acid-modified titanium alkoxides. *J. Non. Cryst. Solids* **2000**, *265*, 276–284. https://doi.org/10.1016/S0022-3093(00)00003-X.
5. Ferraris, S.; Spriano, S.; Miola, M.; Bertone, E.; Allizond, V.; Cuffini A. M.; Banche, G. Surface modification of titanium surfaces through a modified oxide layer and embedded silver nanoparticles: Effect of reducing/stabilizing agents on precipitation and properties of the nanoparticles. *Surf. Coatings Technol*. **2018**, *344*, 177–189. https://doi.org/10.1016/j.surfcoat.2018.03.020.
6. Chen, Z.; Wang, C.; Chen, J.; Li, X. Biocompatible, functional spheres based on oxidative coupling assembly of green tea polyphenols. *J. Am. Chem. Soc*. **2013**, *135*, 4179–4182. https://doi.org/10.1021/ja311374b.
7. Yu, H.; Guo, Z.; Wang, S.; Fernando, G. S. N.; Channa, S.; Kazlauciunas, A.; Martin, D. P.; Krasnikov, S. A.; Kulak, A.; Boesch, C. Fabrication of hybrid materials from titanium dioxide and natural phenols for efficient radical scavenging against oxidative stress. *ACS Biomater. Sci. Eng*. **2019**, *5*, 2778–2785. https://doi.org/10.1021/acsbiomaterials.9b00535.
8. Huang, X.; Wu, H.; Liao, X.; Shi, B. One-step, size-controlled synthesis of gold nanoparticles at room temperature using plant tannin. *Green Chem.* **2010**, *12*, 395–399. https://doi.org/10.1039/B918176H.
9. Bulut, E.; Ozacar, M. Rapid, facile synthesis of silver nanostructure using hydrolyzable tannin. *Ind. Eng. Chem. Res*. **2009**, *48*, 5686–5690. https://doi.org/10.1021/ie801779f.
10. Socrates, G. *Infrared and Raman characteristic group frequencies: tables and charts,* 3rd ed.; Wiley: Chichester, UK, 2004. ISBN 0470093072.
11. Pereira, K.K.Y.; Alves, O. C.; Novaes Jr, A.B.; de Oliveira, F.S; Yi, J.-H.; Zaniquelli, O.; Brandstetter, C.W.; Scharnweber, D.; Variola, F.; Nanci, A.; Rosa, A. L.; de Oliveira, P.T. Progression of Osteogenic Cell Cultures Grown on Microtopographic Titanium Coated With Calcium Phosphate and Functionalized With a Type I Collagen-Derived Peptide. *J. Periodontol.* **2013**, *84*, 1199–1210, https://doi.org/10.1902/jop.2012.120072.
12. de Oliveira, P.T.; Andrade de Oliva, M.; Maximiano, W.M.A.; Sebastião, K.E.V.; Crippa, G.E.; Ciancaglini, P.; Beloti M.M.; Nanci, A.; Rosa, A.L. Effects of a Mixture of Growth Factors and Proteins on the Development of the Osteogenic Phenotype in Human Alveolar Bone Cell Cultures. *J. Histochem. Cytochem.* **2008**, *56*, 629–638, https://doi.org/10.1369/jhc.2008.950758.
13. Stábile, M.F.; Soubelet, C.G.; Albano, M.P.; Rosa, A.L.; de Castro-Raucci, L.M.S.; de Oliveira, P.T. Effect of 64S bioglass addition on sintering kinetic, flexural strength and osteoblast cell response of yttria-partially stabilized zirconia ceramics. *Int. J. Appl. Ceram.* **2019**, *16*, 517–530, https://doi.org/10.1111/ijac.13139.
